# Mitochondrial superoxide dismutase activation with 17 β-estradiol-treated human lens epithelial cells

**Published:** 2008-05-16

**Authors:** Srinivas Gottipati, Patrick R. Cammarata

**Affiliations:** Department of Cell Biology and Genetics, University of North Texas Health Science Center at Fort Worth, Fort Worth, TX

## Abstract

**Purpose:**

17 β-estradiol (17β-E_2_) protects human lens epithelial cells against oxidative stress by preserving mitochondrial function in part via the non-genomic rapid activation of prosurvival signal transduction pathways. The study described herein examined whether 17β-E_2_ also elicits genomic protection by influencing the expression (and activity) of mitochondrial-associated manganese superoxide dismutase (MnSOD) as a possible parallel mechanism by which 17β-E_2_ protects against oxidative stress.

**Methods:**

Virally-transformed human lens epithelial cells (HLE-B3) were pre-incubated with 17β-E_2_, and mRNA or protein lysates were collected over a time course ranging from 90 min to 24 h. Positive expression of lens epithelial cell MnSOD mRNA was determined by semi-quantitative reverse transcriptase polymerase chain reaction (RT–PCR), and its levels were monitored by real-time PCR up to 24 h after 17β-E_2_ administration. Western blot analysis was used to examine the pattern of protein expression as influenced by 17β-E_2_ treatment. MnSOD activity as influenced by 17β-E_2_ was determined by measuring enzymatic activity.

**Results:**

A significant rapid increase in the activity of MnSOD was observed with HLE-B3 cells by 90 min post-bolus addition of 17β-E_2_, which returned to control level by 240 min. Neither an increase in MnSOD mRNA nor in protein expression was detected up through 24 h.

**Conclusions:**

These data demonstrate that 17β-E_2_ rapidly and transiently increases the activity of MnSOD but influences neither its mRNA expression nor its protein expression. The results suggest that (estrogen-activated) MnSOD plays an important role against mitochondrial oxidative stress by diminishing reactive oxygen species, thus promoting cell survival.

## Introduction

Epidemiological studies have indicated a higher incidence of cataract formation in postmenopausal women as compared to men of the same age, suggesting that the absence of estrogens may contribute to their increased risk [1]. The Beaver Dam Eye Study [2] and the Salisbury Eye Study [3] both found a protective association between the use of estrogen and the risk of cataract development. These findings have been further substantiated in studies using rodent models and cell cultures. Using a transgenic mouse model expressing a dominant-negative form of estrogen receptor α, which inhibits estrogen receptor α function, it was demonstrated that female mice spontaneously formed cortical cataracts after puberty, and the disease progressed with age, thereby suggesting that the repression of (nuclear) estrogen action induces cortical cataract [4]. Estrogen treatment diminished the incidence of cortical cataracts in ovariectomized rats treated with methylnitrosourea (MNU) [5]. It has also been reported that estrogen protected lenses against cataracts induced by transforming growth factor-β (TGFβ) in cultured rat lenses [6]. Numerous studies have established that the cytoprotective benefits of estrogen are achieved by its ability to act via both genomic and non-genomic pathways [7].

Cataract is a worldwide leading cause of blindness and is a multifactorial eye disease. While surgical procedures can correct vision loss, this presents a large financial burden on national health care systems mandating the search for pharmaceutical agents that can prevent or delay the onset of cataract [8,9]. Oxidative damage resulting from free radicals and/or H_2_O_2_ is considered to be a major risk factor in the pathogenesis of both age-related and diabetic cataract [10-13]. Elevated levels of H_2_O_2_ have been reported in the aqueous humor of cataract patients, and free radicals and H_2_O_2_ have been implicated in cataract formation [14,15]. Mitochondria are especially sensitive to oxidative stress. H_2_O_2_ can cause the collapse of mitochondrial membrane potential (Δψ_m_) in many cell types including lens epithelial cells, exacerbating free radical production [16,17]. It has been reported that 17 β-estradiol (17β-E_2_) can protect human lens epithelial cells against oxidative stress by preserving mitochondrial function [17]. 17β-E_2_ stabilizes Δψ_m_ in cultured human and bovine lens epithelial cells, acting as a positive regulator of the mitogen-activated protein kinase (MAPK) signal transduction pathway [18]. These effects did not require prolonged exposure to estrogens, suggesting that estrogens are acting at least in part via rapid non-genomic pathways. Studies from our laboratory recently demonstrated that silencing extracellular signal-regulated kinase 2 (ERK2) dramatically increased membrane depolarization compared to non-specific siRNA. That is, ERK2 regulates mitochondrial membrane depolarization, termed, mitochondrial permeability transition (MPT) in human lens epithelial cells, supporting the notion that estrogen-induced activation of ERK2 acts to protect cells from acute oxidative stress. Furthermore, despite the fact that ERK2 plays a regulatory role on mitochondrial membrane potential, it was reported that estrogen-blocked mitochondrial membrane depolarization via an ERK-independent mechanism [19]. Future studies will be aimed at discovering the means by which phosphorylated ERK prevents MPT. Estrogen might directly associate with elements of the MPT pore or indirectly activate/promote phosphorylation of mitochondrial protein components involved in the regulation of the MPT pore, thus opposing the cell death machinery. Such data will undeniably be of great importance to understanding the non-genomic, estrogen-mediated prevention of mitochondrial membrane depolarization.

The study reported herein illustrates the effects of 17β-E_2_ on the expression and activity of mitochondrial-associated manganese superoxide dismutase (MnSOD). A decrease in SOD has been reported in diabetic patients [20]. It has also been reported that MnSOD exerts a protective effect against H_2_O_2_-induced oxidative stress in cultured lens epithelial cell line, SRA 01/04 [21]. Cell cultures made deficient in MnSOD by downregulating the enzyme have been shown to display a pattern of mitochondrial damage, cytochrome C leakage, caspase 3 activation, and increased apoptotic cell death when challenged with a superoxide anion [22].

## Methods

HLE-B3 cells, a human lens epithelial cell line immortalized by SV-40 viral transformation [23], were obtained from Usha Andley (Department of Ophthalmology, Washington University School of Medicine, St Louis, MO). Cells were maintained at 37 °C in Eagle’s minimal essential medium (MEM) supplemented with 20% fetal bovine serum (FBS; Hyclone Laboratories, Logan, UT), 2 mM L-glutamine, nonessential amino acids, and 0.02 g/l gentamicin solution (Sigma Chemical Co, St. Louis, MO) in an environment composed of 5% CO_2_/95% O_2_. All experiments were performed with monolayers of HLE-B3 cells between passages 15–22. To deplete the cell cultures of estrogens, cells were maintained in 20% FBS MEM for 24–48 h then switched to 2% charcoal dextran-stripped FBS (CSFBS-MEM; Gemini Bio-Products, Woodland, CA) for up to 18 h with a final medium change to 0.5% CSFBS-MEM for 24 h.

1,3,5(10) estratrien-3, 17β-DIOL (17β-E_2_) was purchased from Steraloids Inc. (Newport, RI). The hormone was dissolved in 100% ethanol, and stock solutions of hormone were prepared fresh for each experiment and diluted in the culture medium to a working concentration of 1 μM. Control cells received an equivalent aliquot of ethanol. Rabbit polyclonal antibody against human SOD2 (MnSOD) was purchased from Abcam (Cambridge, MA). Rabbit polyclonal antibody against actin and secondary antibodies were purchased from Santa Cruz Biotechnology Inc. (Santa Cruz, CA).

### Reverse transcriptase/polymerase chain reaction

HLE-B3 cells maintained in 75 cm^2^ culture flasks were harvested by scraping and rinsed once with 1X PBS (pH 7.4) and then pelleted by centrifugation at 3000x g for 5 min. Total RNA was extracted using either a TRIzol^®^ Reagent (Invitrogen Corp, Carlsbad, CA) or a Trizol kit (Tel-Test, Friendswood, TX) according to the supplier's protocol. RNA pellets were air dried for 10 min and subsequently treated with Turbo DNA-free (Ambion, Austin, TX) and dissolved in deionized water as per manufacturer’s protocol. The concentration and purity of the RNA preparation were determined by measuring the absorbance of RNA at wavelengths 260 and 280 nm (Hitachi Instruments Inc., Tokyo, Japan). RNA was stored at −80 °C for subsequent experiments.

cDNA was prepared with AMV reverse transcriptase (Promega, Madison, WI) using random hexamer primers (Promega, Madison, WI). RNA was initially denatured at 85 °C for 3 min then placed on ice for 3–5 min. The reaction was performed in a total volume of 20 μl containing 1.0 μg of total RNA, 10 U of AMV reverse transcriptase, 25 ng μl^−1^ random hexamer, 4 μl of 5X AMV reverse transcriptase buffer, 4 μl of 5 mM MgCl_2_, 1 mM dNTPs (Promega, Madison, WI), and 2 U/μl RNasin (Promega, Madison, WI). The reaction was incubated at 42 °C for 45 min. Polymerase chain reaction (PCR) primers were specifically designed for MnSOD using Primer3 (MIT, Cambridge, MA) and synthesized by Sigma Genosys (Spring, TX).

For PCR reactions, 2.5 μl of cDNA from the reverse transcriptase reactions was amplified in a total volume of 50 μl containing 0.2 μM of target gene primers (sense and antisense), 0.75 mM MgCl_2_, 0.2 mM each of dATP, dGTP, dCTP, and dTTP, 1 U of Taq polymerase (Promega, Madison, WI), and 5 μl of 10X PCR buffer (Promega, Madison, WI). Samples were overlaid with 200 μl mineral oil. Amplification was performed on a Perkin Elmer DNA Thermal Cycler 480 (Perkin Elmer, Boston, MA) for 35 cycles. The authenticity of PCR products were confirmed by DNA sequencing (Seqwright, Houston, TX) and by a BLAST search of the sequence through the National Center for Biotechnology Information (NCBI) database (data not shown).

### Real-time polymerase chain reaction

Real-time PCR was performed (Mx3000P Real-Time System; Stratagene La Jolla, CA) with PCR Master Mix (SYBR Green; Stratagene). Each reaction contained 12.5 μl of 2X Master Mix, 500 nM MnSOD forward and reverse primers, and 2.5 ng cDNA from HLE-B3 cells that were treated with estrogen or ethanol (controls) at various time points of 0, 1.5, 3, 6, 12, and 24 h.

Cycle threshold (C_t_) values were normalized to the housekeeper TATA binding protein (TBP), and comparative quantification was performed based on a 2-ΔΔC_t_ calculation method. The specific primers used for MnSOD were 5′-CTG ATT TGG ACA AGC AGC AA and MnSOD 3′-CTG GAC AAA CCT CAG CCC TA, product size 199 bp and for TBP 5′- GAA ACG CCG AAT ATA ATC CCA and TBP 3′-GCT GGA AAA CCC AAC TTC TG, product size 181 bp.

### Cell lysis, electrophoresis, and western blot

After treatments, total cell lysates were collected from HLE-B3 cultures by rinsing adherent cells with ice-cold 1X phosphate buffered saline (PBS), pH 7.4, immediately followed by an addition of lysis buffer (25 mM HEPES, pH 7.4, 0.25 NaCl, 0.5% IGEPAL [NP-40], 0.2% Triton X-100, 1 mM EGTA, 1 mM EDTA, 0.5 mM DTT, 10 mM NaF, 0.1 mM Na_3_VO_4_, and a cocktail of protease inhibitors [Sigma-Aldrich, St Louis, MO]) to the monolayers for 30 min at 4 °C. Lysates were collected, sonicated for 5 s, and sampled for protein concentration using the Bio-Rad protein assay buffer (Bio-Rad Laboratories, Hercules, CA). Laemmli (3X SDS) buffer was added to the lysates, which were subsequently boiled for 3 min, and the proteins were resolved by electrophoresis on 10% SDS-polyacrylamide gels (20 μg protein per lane). Proteins were transferred to nitrocellulose (Scheicher and Schuell, Keene, NH), and the membranes were blocked with 1% bovine serum albumin (BSA) and 0.02% NaN_3_ in Tween-Tris-buffered saline (TTBS) for 15 min. Membranes were probed for 3 h at room temperature and overnight at 4 °C with primary antibodies (see Methods), rinsed in TTBS (4 times for 5 min each time), and incubated in goat anti-rabbit horseradish peroxidase conjugate for 1 h at room temperature. Required concentrations of antibodies were determined according to the manufacturer’s protocols. Membranes were again rinsed in TTBS (4 times for 5 min each time), and proteins were detected using a SuperSignal West Pico Chemiluminescent kit from Pierce (Rockford, IL). Probed membranes were exposed to Kodak BioMax Light Film (Kodak Scientific Imaging, Rochester, NY).

### Superoxide dismutase enzyme activity assay

For MnSOD activity evaluation, cells were lysed, collected with a rubber policeman, and sonicated in cold 20 mM HEPES buffer, pH 7.2 (EGTA 1 mM, 210 mM mannitol, and 70 mM sucrose) followed by centrifugation at 1500x g for 5 min at 4 °C. The supernatant was subsequently centrifuged at 10,000x g for 15 min at 4 °C. The supernatant was evaluated for MnSOD activity evaluation using a Superoxide Dismutase Assay kit (#706002; Cayman Chemicals Inc., Ann Arbor, MI) according to the manufacturer's protocol. The assay kit is designed to measure total superoxide dismutase (SOD) activity (cytosolic and mitochondrial). Separation of the two enzyme activities was achieved as directed by the manufacturer's protocol. Briefly, the 1500x g supernatant of the cell lysate was re-centrifuged at 10,000x g for 15 min at 4 °C. The resulting supernatant contains the cytosolic enzyme (Cu/Zn-SOD), and the pellet contains the the mitochondrial enzyme (MnSOD). The pellet was homogenized. The addition of 1–3 mM potassium cyanide to the assay inhibits both Cu/Zn-SOD and extracellular SOD, resulting largely in the detection of MnSOD alone.

### Statistical analysis

For the MnSOD enzyme assay, significant differences between groups were determined by an independent sample Student’s *t*-test (2-tailed) using SPSS version 12.0 for Windows. For quantitative polymerase chain reaction (QPCR), normalized expression data were compared by two-way ANOVA in all cases. For all experiments, data are reported as mean±SEM or SD as indicated, and p values <0.05 were considered significant.

## Results

### Reverse transcriptase polymerase chain reaction detection of manganese superoxide dismutase mRNA

**Figure 1:**
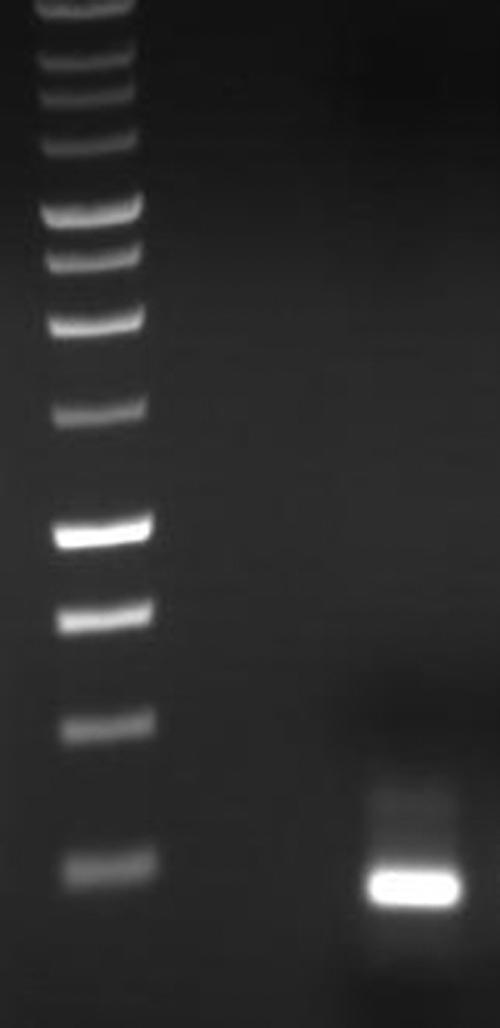
Reverse transcriptase polymerase chain reaction expression of manganese superoxide dismutase in HLE-B3 cells. Total RNA was extracted from confluent HLE-B3 cells and subjected to RT–PCR for MnSOD. The cDNA product yielded one band at the correct predicted molecular weight (199 bp). The authenticity of PCR products was verified by DNA sequencing and a BLAST search of the sequence (refer to Methods).

Total RNA was extracted from cultured HLE-B3 cells and subjected to RT–PCR for the detection of mitochondrial-associated MnSOD. The resulting cDNA product yielded one band at the correct predicted molecular weight (Figure 1).

### Effect of 17β-E_2_ on manganese superoxide dismutase mRNA expression

**Figure 2:**
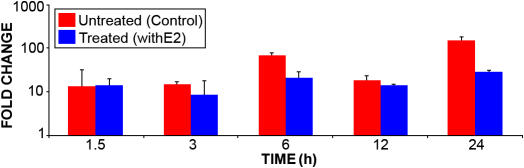
Real-time polymerase chain reaction expression of manganese superoxide dismutase in HLE-B3 cells. HLE-B3 cells were incubated with 1 μM 17β-E_2_ for 0, 1.5, 3, 6, 12, and 24 h. The expression of MnSOD mRNA was analyzed by quantitative real-time PCR. According to the two-way ANOVA, there were no statistically significant changes in MnSOD mRNA at any time point after estrogen treatment compared to the control (untreated). Data are expressed as mean±SD values. p<0.05 (n=3) was considered significant.

HLE-B3 cells were maintained in 75 cm^2^ culture flasks in 20% FBS MEM for 24–48 h then switched to 2% CSFBS-MEM for up to 18 h with a final medium change to 0.5% CSFBS-MEM for 24 h. Cells were incubated with either 1 µM of 17β-E_2_ or ethanol-substituted control for 1.5, 3, 6, 12, and 24 h. Each collected time point was compared to its parallel control population of cells, which had not been treated with 17β-E_2._ MnSOD mRNA was analyzed by real-time PCR (Figure 2). The level of expressed MnSOD with 17β-E_2_ treatment was comparable to its non-treated counterpart. The addition of 17β-E_2_ did not elicit any statistically significant change in the levels of expressed MnSOD over the duration of the time course.

### Effect of 17β-E_2_ on MnSOD protein expression

**Figure 3:**
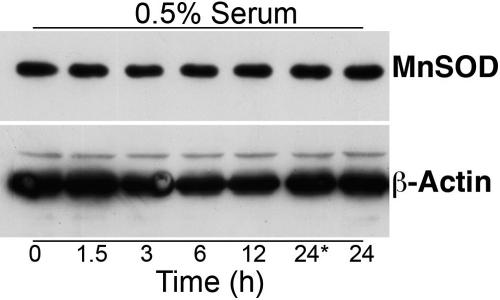
Western blot analysis of manganese superoxide dismutase expression in HLE-B3 cells. Total cell lysates were collected from HLE-B3 cells grown in 0.5% serum and stimulated by exposure to 1 μM 17β-E_2_ for 0, 1.5, 3, 6, 12, and 24 h. There was no change in the expression of MnSOD at any of the time points. The time points at 0 and 24* hours did not receive estrogen.

HLE-B3 cells were maintained in 0.5% CSFBS-MEM (see Methods). Cells were incubated with 1 µM 17β-E_2_ for 1.5, 3, 6, 12, and 24 h. The 0 h and one of the 24 h time points (indicated as 24* in Figure 3) served as the controls and received an equivalent ethanol concentration without estrogen. MnSOD protein expression was monitored by western blot analysis. No demonstrable change in the level of protein expression of MnSOD was detected over the entire time course of collected samples.

### Effect of 17β-E_2_ on manganese superoxide dismutase activity

Two experiments using separate cell populations were performed with the cells maintained in 0.5% serum as described above. Cell cultures were incubated with 17β-E_2_ for 30 min and 90 min (Table 1) or 90 min and 6 h (Table 2) after which MnSOD activity was immediately determined. No statistically significant difference was observed in the samples treated with 17β-E_2_ for 30 min in that there was no observed increase in MnSOD activity with estrogen treatment relative to the control cells not treated with estradiol (Table 1). The 90 min incubation with 1 µM 17β-E_2_ significantly increased MnSOD activity compared to the untreated controls, and this was repeated with two independent experiments (see Table 1 and Table 2). A second, independent experiment was performed over a longer duration of estradiol treatment (Table 2). Lens cells were likewise maintained in 0.5% serum, but this time, they were incubated with 17β-E_2_ for 90 min and 6 h. As with the first set of treated cells (Table 1), 90 min of incubation with 1µM 17β-E_2_ increased MnSOD activity compared to its untreated control counterpart (Table 2). No statistically significant difference was observed between the samples treated with 17β-E_2_ for 6 h and its untreated counterpart (Table 2), and more importantly, MnSOD activity had returned to a level equivalent to that observed with 30 min of treatment (Table 1).

**Table 1:** 17β-E_2_ upregulates manganese superoxide dismutase activity.

**Time**	**U/ml**		**Time**	**U/mg protein**	
	**Control**	**(+) 17b E2**		**Control**	**(+) 17b E2**
30 min	54.9±6.1	67.7±6.5	30 min	24.5±5.3	29.4±5.1
90 min	66.4±9.1	104±12.1*	90 min	27.4±3.8	49.6±7.4*

HLE-B3 cells maintained in 0.5% serum were exposed to 1 μM 17β-E_2_ for 30 and 90 min (n=8, from two individual cell populations±SEM). The control represents cells that were not treated with 17β-E_2_. Note the significant increase in activity (asterisk) detected with the 90 min incubation treated with 17β-E_2_. The MnSOD activity (refer to Methods) was expressed as units/ml (left panel) or units/mg protein (right panel). n=8 from two independent cell populations; the asterisk indicates statistical significance as determined by Student’s *t*-test.

**Table 2:** 17β-E_2_ does not upregulate manganese superoxide dismutase activity after 6 h.

**Time**	**U/ml**		**Time**	**U/mg protein**	
	**Control**	**(+) 17b E2**		**Control**	**(+) 17b E2**
90 min	20.4±4.4	91±10.8*	90 min	11.6±3	62±8*
6 h	45±12	49±6	6 h	30±8	33±5

A second independent experiment with a fresh cell population was repeated over an extended time course with HLE-B3 cells maintained in 0.5% serum and exposed to 1 μM 17β-E_2_ for 90 min and 6 h (n=8, from two individual cell populations±SEM). The control represents cells that were not treated with 17β-E_2_. Note the significant increase in activity (asterisk) detected with the 90 min incubation treated with 17β-E_2_ and the return to equivalent control activity levels by 6 h of treatment (compare 6 h control versus 6 h +17β-E_2_). n=8; the asterisk indicates statistical significance as determined by Student’s *t*-test.

### Discussion

Cellular damage caused by the accumulation of reactive oxygen species (ROS) has been implicated in many disease processes, especially in age-related disorders. Lens cells are particularly subject to oxidative stress due to photooxidative processes and are consequently prone to cellular oxidative damage [24]. ROS can cause oxidative modifications of several cellular constituents such as proteins, cytoskeletal elements, membrane sulfhydryls, glutathione levels, and alterations in transport systems in the epithelium. These changes are in turn thought to result in lens opacification and nuclear cataracts [21,25,26]. Mitochondria have been found to be especially susceptible to oxidative damage. Oxidant damage to the mitochondria can cause the release of calcium, protein oxidation, depletion of ATP, lipid peroxidation, and DNA damage [27]. The lens is equipped with antioxidant enzymes, which can prevent the toxic effects of free radicals. Superoxide anion is dismutated by the enzyme, superoxide dismutase (SOD), to yield H_2_O_2_, which is then eliminated by glutathione peroxidase and catalase [28]. There are three isoforms of this enzyme, the cytosolic-CuZnSOD (SOD1), mitochondrial superoxide dismutase MnSOD (SOD2), and the extracellular SOD (ecSOD).

Various studies using tissue culture and animal models have demonstrated the beneficial effects of estrogen in the lens including the prevention or delay in the onset of cataract formation [4-6]. The classic model that describes the action of 17β-E_2_ illustrates 17β-E_2_ binding to a receptor and the complex then being transferred to specific promoter-regulatory DNA elements, which prompt nuclear gene transcription activation or repression and subsequent protein synthesis [7,29]. However, non-genomic actions of steroid hormones have been described. Recent work from this laboratory has focused on investigating such alternative non-genomic pathways to explain the rapid cytoprotective effects of estrogens [17-19].

With respect to genomic response, there are several recent studies with opposing results relating to the effect of estrogen on the expression and activity of MnSOD. Using cultured vascular smooth muscle endothelial cells, it has been reported that the expression and the activity of MnSOD is enhanced by estrogens. Pre-incubation with 1 µM 17β-E_2_ elevated the expression of MnSOD mRNA after 12 h. The protein level of MnSOD was enhanced after 14 h followed by an increase in MnSOD activity 24 h post-incubation. Furthermore, these effects on MnSOD expression and activity were reported to be mediated by the activation of estrogen receptors as the increased expression of MnSOD mRNA was blocked by incubation with estrogen receptor antagonist, ICI 182,780 [30]. In another study, it was reported that the human breast carcinoma cells, MCF-7, when incubated with physiologic concentrations of estrogen (0.02 nM) for a period of 48 h demonstrated an increase in the expression of MnSOD mRNA, and the upregulation of MnSOD was linked to MAPK and nuclear factor kappa (NFκB) signaling pathways [31]. It was recently reported that 17β-estradiol significantly reduced the rate of superoxide production in a receptor dependent manner using rat pheochromocytoma cells (PC-12 cells). Gonadectomized animals were treated with testosterone, dihydrotestosterone, and estrogen to assess the in vivo effects of gonadal hormones on brain mitochondrial oxidative stress in male and female rats. Only estrogen decreased brain mitochondrial ROS production in vivo. However, in apparent opposition to the two studies reported above [30,31], estrogen was reported to increase MnSOD activity levels without affecting the protein levels of MnSOD in mitochondria [32]. This observation has also been independently substantiated in that estrogen upregulates the activity of MnSOD in a rapid manner (10–20 min) but does not alter protein expression of MnSOD in the mitochondria [33]. Thus, 17β-E_2_ seems to enhance mRNA transcription, protein levels, and activity of MnSOD in some cell systems while only increasing MnSOD activity in others. Furthermore, it is noteworthy to point out that there is considerable variability as to the timeframe required (i.e., minutes versus hours) for the estrogen-mediated response to increase MnSOD activity.

The study described herein also examined the effect of estrogens on the expression and activity of MnSOD as part of our preliminary investigation into possible cytoprotective genomic responses by estrogen in cultured human lens epithelial cells. With the cultured lens epithelium system, no change in the expression of MnSOD mRNA was observed over 24 h post-incubation with estradiol according to results obtained by real-time PCR (Figure 2). HLE-B3 cells treated with 17β-E_2_ over a similar time course also showed no alteration in protein expression for MnSOD according to western blot analysis (Figure 3). However, a rapid and transient increase in the activity of MnSOD was observed where the activity peaked in 90 min (Table 1 and Table 2) and returned to control levels within 6 h (Table 2). This rapid and transient increase in MnSOD activity unaccompanied by a coupled change in its mRNA and protein expression argues for non-genomic action of estradiol with cultured human lens epithelial cells.

What then is the potential mechanism and beneficial effects of a mitochondrial MnSOD response mechanism that senses a rapid (and as a result, transient) “spike” in reactive oxygen species (ROS)? The accumulation of ROS, produced by a wide variety of exogenous chemical and metabolic processes, can play a dual role in biologic systems dependent on the relative level of ROS intracellular concentration. ROS can either be beneficial or harmful [34]. At low concentrations, ROS can act as second messengers in signaling cascades leading to the induction of mitogenic responses whereas at higher concentrations, ROS cause damage to various cellular structures including lipids, proteins, and nucleic acids [35]. Mitochondria are considered to be a major source of ROS, which include superoxide anion (O_2_^-^), H_2_O_2_, and the hydroxyl free radical. These compounds may play key roles as signaling molecules regulating mitochondrial dysfunction and subsequent apoptotic events. It has been reported that exposure to estrogen stimulates the rapid production of intracellular ROS in human umbilical vein endothelial cells [36]. Estrogen-induced mitochondrial reactive oxygen species act as signal-transducing messengers [37]. Studies have shown that estrogen causes an increase in mitochondrial-associated calcium [38]. Ca^2+^ can further enhance the dislocation of cytochrome c from the inner mitochondrial membrane by competing with cardiolipin binding sites, such competition results in the blocking of complex III, which would enhance ROS generation [39]. An increased mitochondrial formation of ROS triggers the intrinsic pathway of apoptosis by increasing the permeability of the outer mitochondrial membrane through the opening of the mitochondrial permeability transition pore [40]. MnSOD preserves mitochondrial function by regulating the sensitivity of the permeability transition pore to ROS [41]. In our study, the endogenous accumulation of reactive oxygen species (ROS) was assessed in HLE-B3 cells treated with estrogen by loading cells with H_2_DCF-DA, which upon oxidation in the presence of ROS transitions to the fluorescent compound, 2,7 dichlorofluorescin (DCF). A statistically significant increase in ROS was observed only after three days in estrogen-treated cells relative to parallel control cell cultures.

Data presented herein supports the notion that the bolus addition of 17β-E_2_ causes a transient increase in the production of ROS in HLE-B3 cells but at the same time constitutively induces MnSOD activity, which quenches the ROS formed in the mitochondria and preserves mitochondrial function. Chen et al. [42] have demonstrated that “a low level of reactive oxygen species plays an important role in host defense and mediating mitogen-stimulated cell signaling.” This group has shown that the endogenous generation of ROS initiates redox signaling, resulting in, among other things, the activation of mitogen-activated protein kinases (MAPKs). To that end, it is noteworthy to mention that we have previously reported that cell death induced by H_2_O_2_ in cultured human lens epithelial cells is associated with the accumulation of intracellular ROS, the collapse of the mitochondrial membrane potential (Δψ_m_), and the depletion of ATP. When estradiol is present, it requires more Ca^2+^ to induce comparable Δψ_m_ collapse or conversely, under comparable Ca^2+^ loading, a larger portion of the mitochondrial population retains its membrane potential in the presence of estradiols [17]. It has also been shown that 17β-E_2_ acts as a positive regulator of the survival signal transduction pathway, MAPK, which, in turn, acts to stabilize Δψ_m_ [18,19].

Our conceptual framework regarding the protective mechanism(s) activated by 17β-E_2_ is based on the premise that cytoprotection will prove to be complex and multi-faceted with both non-genomic and genomic aspects. We support the notion that the estrogen-driven responses driving mitochondrial protection against membrane potential loss are likely to prove to be dynamic. The protective stabilization of mitochondrial membrane potential by estrogens may be attributed to the consolidation of several mechanisms of action working in concert. To that end, we can now report that there is a coupled duality to the mechanism of estradiol’s cytoprotective action against acute oxidative stress. Estradiol administration to HLE-B3 cells initiates a rapid increase in intracellular (mitochondrial) ROS and transiently increases MnSOD activity, which immediately acts to lower the ROS concentration before mitochondrial damage might ensue. At the same time, estradiol prompts a signaling cascade culminating in the activation of the ERK/MAPK pathway, which exerts a positive effect by attenuating the extent of depolarization of mitochondrial membrane potential in the face of acute oxidative stress and thereby preventing entry into the cell death pathway.

Other mitochondrial protective mechanisms against oxidative stress are likely. The restraint of Δψ_m_ collapse might be explained by a repression of Ca^2+^ uptake into the mitochondria, increased tolerance to mitochondrial calcium sequestration, increased Ca^2+^ efflux from the mitochondria, increased resorption of Ca^2+^ into endoplasmic reticulum, and/or increased efflux of Ca^2+^ via the plasma membrane. An estrogen redox cycle has been proposed [43], which may control glutathione and NAD(P)H flux, and in conjunction with “classic” antioxidant responses [44], the the redox cycle may be acting as a defense mechanism against reactive oxygen species. Recently, it has been reported that protein kinase C epsilon (PKCε) is activated by hypoxia, and this results in the activation of the mitochondrial protein, cytochrome c oxidase IV subunit (CytCOx). This potentially provides another mechanism to protect the lens from mitochondrial damage under the naturally hypoxic conditions observed in this tissue [45].
